# Neutralization of IL-15 abrogates experimental immune-mediated cholangitis in diet-induced obese mice

**DOI:** 10.1038/s41598-018-21112-7

**Published:** 2018-02-15

**Authors:** José L. Reyes, Danielle T. Vannan, Tina Vo, Aliya Gulamhusein, Paul L. Beck, Raylene A. Reimer, Bertus Eksteen

**Affiliations:** 1 Snyder Institute for Chronic Diseases, Cumming School of Medicine, University of Calgary, Calgary, Alberta Canada; 2 Laboratorio de Inmunología Experimental y Regulación de la Inflamación Hepato-Intestinal, UBIMED, FES Iztacala UNAM, Estado de México, Mexico; 3 Genomic Hepatobiology Laboratory, Mayo Clinic, Rochester, Minnesota USA; 4 Department of Biochemistry & Molecular Biology, Cumming School of Medicine, University of Calgary, Calgary, Alberta Canada; 5 Faculty of Kinesiology, University of Calgary, Calgary, Alberta Canada; 6 Aspen Woods Clinic, Calgary, Alberta Canada

## Abstract

Obesity is a global epidemic affecting chronic inflammatory diseases. Primary sclerosing cholangitis (PSC) is a chronic cholestatic liver disease that can occur as an extraintestinal manifestation of inflammatory bowel disease (IBD). Previously we reported that patients with PSC who are obese have a higher risk of advanced liver disease. Currently it is unknown how obesity accelerates or worsens PSC. We evaluated the progression of PSC in an antigen-driven cholangitis mouse model of diet-induced obesity. Obesity was induced in our murine model of immune-mediated cholangitis (OVAbil). OVAbil mice were fed standard chow or high-fat/sucrose diet for twelve weeks followed by induction of biliary inflammation by OVA-specific T cell transfer. Histopathological damage in portal tracts was scored and serum collected. Neutralizing antibodies against IL-15 were administered daily until study termination. Obese mice developed exacerbated liver inflammation and damage. Immune cell phenotyping in liver revealed greater numbers of neutrophils and CD8+ T cells in obese mice. Higher levels of cytokines and chemokines were found in obese mice with cholangitis. Immuno-neutralizing antibodies against IL-15 greatly attenuated cholangitis in obese mice. Obesity exacerbated experimental PSC in part by overproduction of IL-15. Timely targeting of IL-15 may slow the progression of PSC.

## Introduction

The dramatic increase in the world-wide prevalence of obesity is recognized as a risk factor for development of numerous complicated co-morbidities^1^. Research has uncovered an important role for adipose tissue not only as an energy-storage organ but also as a potent source of inflammatory mediators including leptin, TNF-α and macrophage-attractant chemokines (MCP1, MIP-1 and MIF)^2^. During obesity, the release of proinflammatory cytokines and chemokines from adipose tissue may re-program resident immune cells towards an augmented inflammatory profile and may result in the recruitment of immune cell populations that are otherwise under-represented^3^. Consequently, the development of chronic, low-grade inflammation associated with obesity may exacerbate inflammatory diseases in other tissues and organs^4^.

Primary sclerosing cholangitis (PSC) is a chronic cholestatic liver disease characterized by the progressive destruction of bile ducts that can lead to portal hypertension^5^. The pathogenesis of PSC remains unknown however an autoimmune origin is widely accepted^5^. A close relationship exists with inflammatory bowel disease (IBD) as PSC is considered one of the extraintestinal manifestations of IBD^3^. Up to 80% of patients with PSC also have IBD whereas approximately 7% of IBD patients may develop PSC^6,7^. Unfortunately, no effective treatment strategies exist and the persistent liver inflammation associated with PSC can lead to fibrosis requiring liver transplantation or the development of cholangiocarcinoma (CC)^8^. Continued examination of genetic and environmental factors such as diet and lifestyle triggering chronic, uncontrolled inflammation is required to understand the pathogenesis of PSC and for the development of effective therapeutics.

We have previously identified a subpopulation of patients based on body mass index (BMI) who are overweight (BMI 25–30) or obese (BMI >30) with PSC that had more advanced fibrosis at presentation and more rapid fibrosis progression as measured by ultrasound transient elastography (Fig. S1)^9^. However, the link between obesity and more severe PSC has yet to be examined. In this current study we explored the impact of diet-induced obesity during the development of cholangitis by using an antigen-specific murine model of PSC. In addition we identified IL-15 in our model as a critical factor driving inflammation and tissue damage within the livers of obese mice with cholangitis. IL-15 has been linked to the promotion of lipolysis and energy expenditure while at the same time is responsible for the maintenance and survival of T cells and NK cells^10,11^. To date, the role of IL-15 in PSC and biliary diseases has been poorly examined. Therefore we evaluated the neutralization of IL-15 in our murine model to gain greater insight into the underlying pathways triggering or accelerating PSC in individuals with obesity in order to identify potential novel therapeutic targets.

## Methods

### Experimental model of biliary damage

The study protocol was approved by the University of Calgary Animal Care Committee and conformed to the *Guidelines for the Care and Use of Laboratory Animals*. Mouse studies were carried out using a modified protocol of the OVAbil model previously described^12^. Briefly, C57BL/6 mice that express ovalbumin peptide (aminoacids 139–385) in a cholangiocyte-restricted manner (OVAbil) were obtained from Dr. Marion G. Peters and used as recipient mice. To induce biliary inflammation, MHC I- and MHC II-restricted OVA-specific T cells (OT) were transferred from the lymphocyte fraction recovered by Percoll gradient from the spleens of donor mice. Recipient OVAbil mice were given 10^7^ cells from each OT I and OT II donor mice via intraperitoneal (i.p.) injection. Five or 10 days after transfer, mice were sacrificed under deep anesthesia and degree of disease was assessed.

### Obesity induction model

Seven to eight week-old male and female OVAbil mice were fed standard chow (SC; 3.8 kcal/g) or a custom high-fat/high-sucrose diet (HFD; 4.6 kcal/g) for twelve weeks (Dyets Inc, USA)^13^. Weight gain was recorded weekly and circulating leptin was measured as a putative marker of increasing adiposity in HFD fed mice.

### Circulating cytokines, chemokines and liver enzyme determination

At indicated time points (before T cells transfer and 5 or 10 days post transfer) mice were anesthetized (Isoflurane) and whole blood was collected via cardiac puncture. Blood samples were centrifuged and serum was stored at −80 °C for future analysis. Alanine aminotransferase (ALT) levels were measured by Calgary Laboratory Services (Calgary, AB, Canada). Quantification of cytokines and chemokines was conducted as reported using a commercial mouse cytokine 31-plex Discovery Assay (Eve Technologies, Calgary, AB, Canada)^14^. The analytes included: eotaxin, G-CSF, GM-CSF, IFN-γ, IL-1α, IL-1β, IL-2, IL-3, IL-4, IL-5, IL-6, IL-7, IL-9, IL-10, IL-12(p40), IL-12(p70), IL-13, IL-15, IL-17, IP-10, KC, LIF, MCP-1, MIG, MIP-1α, MIP-1β, MIP-2, RANTES, TNF-α and VEGF. Briefly, 31 analytes were simultaneously tested in a single well using multiplexing LASER Bead Technology and colour-coded polystyrene beads. Using dual-laser technology and flow cytometry the analyte can be identified based on the bead colour whereas the concentration of analyte present is quantified by the fluorescent intensity using a standard curve. Values are presented in pg/ml.

### Histopathology scoring

At sacrifice liver tissue was excised and rapidly fixed in 10% neutral-buffered formalin for histological assessment. To assess bile duct (BD) inflammation and damage, five portal tracts were randomly scored as follows: inflammation (0–3) 0, no visible inflammatory cells; 1 low number of inflammatory cells; 2 mild inflammation; 3 severe inflammation. BD damage (0–2) 0 BD architecture preserved; 1 loss of typical BD architecture (deranged with infiltrating cells displacing cholangiocytes); 2 absent BD.

### Characterization of immune cell populations in liver via flow cytometry

Fresh liver tissue was placed in cold, sterile PBS with 10% FBS and 2 mM EDTA and immediately homogenized using a gentleMACS tissue dissociator (Miltenyi Biotec Inc, Germany). Tissue homogenate was filtered through a 60 micron mesh to achieve a single cell suspension. Following centrifugation, cells were layered on a 33%/77% Percoll gradient and spun at room temperature at 600 rcf for 20 minutes (GE Healthcare Bio-Sciences AB, Uppsala Sweden). Leukocytes were removed from the gradient, pelleted and resuspended in cold FACS buffer (PBS, 0.5% BSA and 2 mM EDTA) followed by incubation with the following fluorescent-conjugated antibodies: Panel 1; APC/Cy7-Ly6C, PO-CD11b, PE/Cy7-F4/80, PE-Ly6G, FITC-CX3CR1, PerCP-CCR5, APC-CCR1. Panel 2; APC-CD3. PB- CD4, PO-CD8, PE-NK1.1. Data were acquired on a FACS Aria II (BD Biosciences) and analyzed with Kaluza software (Beckman Coulter, USA).

### Gene expression analysis by real-time quantitative PCR

Transcripts of canonical markers of fibrosis were assayed in liver tissue. Briefly, small pieces (~20 mg) of liver tissue were homogenized in TRizol® reagent (Invitrogen, USA) and total RNA was extracted by the chloroform method. The concentration of total RNA was quantified by NanoDrop 2000 (Thermo Fisher Scientific Inc, Mississauga, ON, Canada) followed by reverse transcription using the RT2 first strand cDNA synthesis kit for RT-PCR (Qiagen, Toronto, ON, Canada). The resultant cDNA was amplified using primers generated through the Universal ProbeLibrary (Roche Diagnostics, Laval, QC, Canada) and synthesized by the University of Calgary Core DNA Services (Calgary, AB, Canada). A StepOne Real-Time PCR System was used for the real-time PCR reactions (Thermo Fisher Scientific Inc, Mississauga, ON, Canada) and FAM detection probes (Roche Diagnostics, Laval, QC, Canada). The following primer sequences were used: α-smooth muscle actin (*sma*) 5′-CTCTCTTCCAGCCATCTTTCAT-3′, 5′-CGTAGGTGCTTTGGTGGATAT-3′; tissue inhibitor of matrix metalloproteases -1 (*timp1*) 5′-GCAAAGAGCTTTCTCAAAGACC-3′, 5′- TCACAAAGGGACAAATAGATAGGGA-3′; type 1 collagen (*col1a1*) 5′ CATGTTCAGCTTTGTGGACCT-3′ 5′- TGTAGGGACTTCAGTCGACG-3′; β-actin (housekeeping) 5′-CTAAGGCCAACCGTGAAA-3′, 5′-AGGGACATACGGAGACCA-3′.

### Neutralization of IL-15

In order to neutralize IL-15 bioactivity, mice were treated with anti-IL-15 antibodies (clone GRW15PLZ, eBioscience USA) as previously reported^15^. Briefly, 5 µg of anti-IL-15 or isotype-matched antibodies were injected i.p. daily from day 4 to day 9 post- OVA-specific T cell transfer and disease outcome was assessed on day 10 in experimental groups.

### Data presentation

Data are shown as mean ± SE. Differences between mouse groups was determined by one-way ANOVA followed by Tukey’s posthoc for multiple comparisons or when indicated student’s *t* test. p < 0.05 was accepted as significantly different.

### Data availability

All data generated or analyzed during this study are included in this published article (and its Supplementary Information files).

## Results

### Exposure to high-fat/sucrose diet results in obesity in mice

Experimental groups fed either SC or HFD showed clear changes in bodyweight and leptin secretion over time. Given the male-dominant phenomenon in patients with PSC we started our project by studying female and male mice separately. In terms of bodyweight, we did not see significant differences between sexes after the 12 week-diet administration. Female mice fed SC gained 27.3% (+5.8 g) of the initial weight and female mice fed a HFD gained 49.3% (+11.5 g) of initial weight, whereas male mice fed SC gained 20% of initial weight (+5.3 g) and male mice fed HFD gained 46.6% (+13.8 g). Thus, other than natural sex-specific bodyweight differences we observed similar weight gain in both male and female mice (Fig. S2A).

As leptin is secreted in proportion to body fat by adipocytes we used leptin levels as an indirect measurement of changes in adiposity in the mice. We observed a dramatic difference in serum leptin levels between SC and HFD fed animals over the 12-week period (Fig. S2B). Interestingly, when comparing leptin levels between female and male within the HFD group, we found significant differences on week 6 post-diet (i.e. males had higher levels of leptin than female counterparts), however under the long-term feeding regime leptin concentrations in female mice reached comparable levels to male mice on week 12 (Fig. S2B). The fact that female mice exhibited similar levels of leptin upon long-term HFD feeding compared to male mice suggests that this may be true for additional inflammatory cytokines and may account in part for losing sex differences in our mouse studies. Thus, mouse results are presented as SC group *versus* HFD group given no differences between male and female groups were observed.

### Obese mice developed exacerbated biliary damage

**Figure 1:**
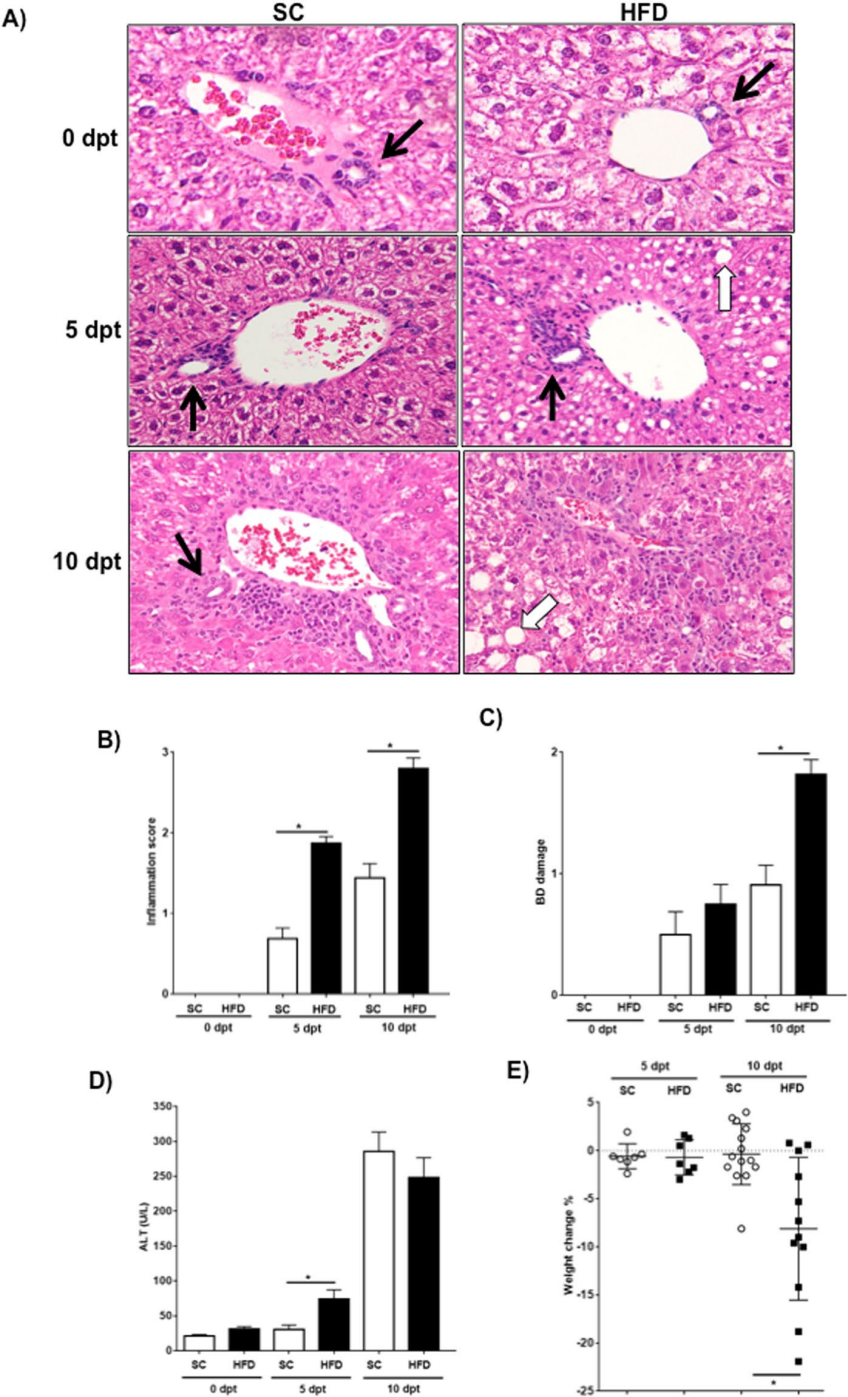
Mice fed HFD presented heightened BD destruction. After twelve weeks of feeding either SC or HFD, following adoptive T cell transfer, mice developed antigen-driven liver injury. Panel (A) Shows representative H&E-stained sections of portal tracts at indicated times after OVA-specific T cell transfer. Black arrows identify bile ducts within the liver tissue. Quantification of inflammatory extent and BD damage is seen in panel (B) and (**C**), respectively. Circulating levels of alanine transaminase (ALT) were detected in serum samples (**D**). Weight change was recorded at indicated times and is shown in panel (E). Data are mean ± SE from 2–3 independent experiments (n = 8–12), where *p < 0.05. dpt; days post transfer.

Diet-induced obese OVAbil mice were employed to assess the relationship between obesity and experimental PSC. Following obesity induction, HFD fed OVAbil mice developed macromolecular steatosis as evident by the accumulation of large lipid droplets within hepatocytes while the bile ducts (BDs) exhibited preserved architecture prior to OVA-T cell transfer (Fig. 1A). Liver sections from mice fed SC revealed uniform liver parenchyma with well-defined bile BDs before the transfer of OVA-T cells (Fig. 1A). Five days following adoptive transfer, increased periportal infiltrating cells were seen in HFD fed OVAbil mice compared to chow fed mice that displayed few infiltrating inflammatory cells in the portal tracts and preserved BDs (Fig. 1A). At the peak of disease (day 10 post-transfer), the majority of the portal tracts in HFD OVAbil mice were reduced and most of the BDs were absent whereas mice fed SC showed increased size in periportal inflammatory foci resulting in reduced portal tract space but still visible BDs (Fig. 1A). The degree of inflammation (p = 0.001) and BD damage was significantly increased in HFD fed mice compared to SC fed mice after 10 days post transfer (p = 0.0002, Fig. 1B and C). Using ALT serum levels as a putative liver damage indicator, we observed a trend for elevated ALT levels at the peak of the disease as compared to baseline levels (before transfer of OVA-specific T cells) while there were no significant differences between SC and HFD mice (Fig. 1D). However, unlike SC mice, significant weight loss was observed in the obese mice (−0.3% vs −8.1% of initial weight in average, respectively) (Fig. 1E).

### Diet-induced obesity dampens re-appearance of Ly6C^lo^ macrophages and accelerates the accumulation of neutrophils

**Figure 2:**
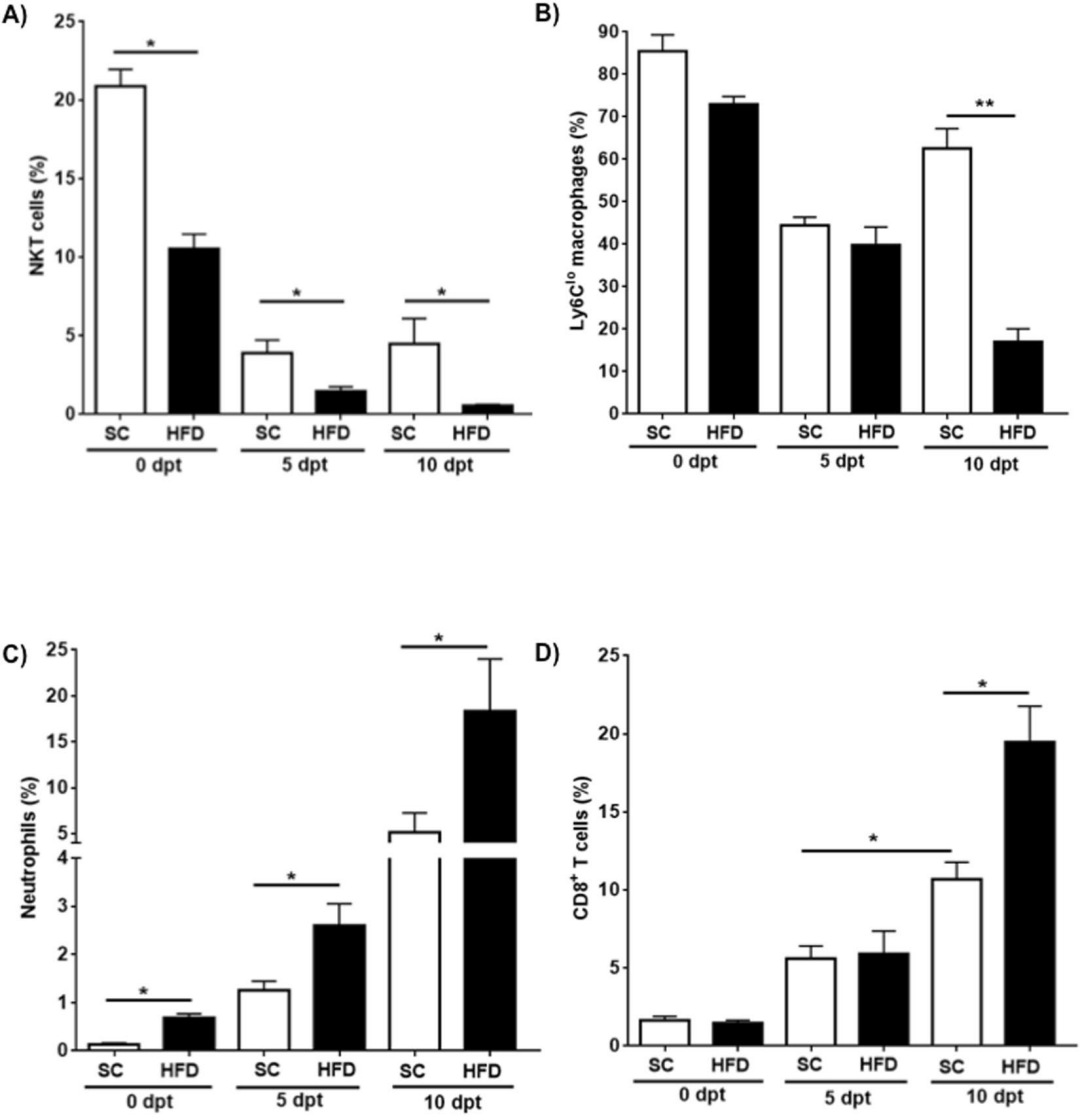
Obese mice showed accelerated loss of NKT cell numbers, impaired ability to repopulate liver with Ly6C^lo^ macrophages, increased infiltration of neutrophils and cytotoxic T cells after liver injury. Following the gating strategy presented in supplemental materials we determined the percent of infiltrating CD4^+^ NKT cells. In panel (A), a graphical representation of % cells found at indicated time points is shown where a significantly accelerated loss of this population was observed. In panel (B), the bar graph shows the percent of Ly6C^lo^ macrophages (restorative macrophages) detected in livers from experimental groups over the time. This population tended to decrease five days after transfer of pathogenic T cells in non-obese mice but exhibited a significant recovery on day 10, the latter was impaired in obese mice. (**C**) Leukocytes recovered from livers were identified as neutrophils (CD11b^+^Ly6G^hi^ SSC^hi^) and (**D**) cytotoxic T cells (CD3^+^CD4^-^CD8^+^) and numbers of these populations are graphically represented in panel C and D, respectively. Both populations steadily increased over the time. Data are shown as mean ± SE of 2–3 independent experiments with same results. *p < 0.05 when compared to time matched counterparts. dpt; days post transfer.

To the best of our knowledge this is the first time experimental PSC has been examined in the context of obesity. In order to further understand the exacerbated disease phenotype in obese OVAbil mice we characterized infiltrating immune cells in the liver using flow cytometry. The specific gating strategy is shown in supplemental data (Fig. S3A). Macrophages are one of the most studied immune cell populations in liver. Others and we have reported gene-profiling data of inflammatory and restorative macrophage subpopulations during liver injury processes underscoring important differences between these cell types^16,17^. We aimed to determine changes in this population in the context of obesity before and after induction of immune-mediated cholangitis. Macrophage populations were broadly subdivided based on Ly6C antigen expression. Upon gating CD11b^+^ myeloid cells, an apparent segregation of F4/80+ Ly6C^hi^ and Ly6C^lo^ macrophage subpopulations was observed (Fig. S3C). Others have reported this same strategy to classify newly recruited (Ly6C^hi^) *versus* long-term resident (Ly6C^lo^) populations^17^. The majority (>80%) of the Ly6C^+^ populations were CCR2^+^ with a few CX3CR1^+^ (Fig. S4). Mice fed SC initially presented an abundant Ly6C^lo^ macrophage population (85% of myeloid CD11b^+^ cells), which upon liver injury induction (i.e. 5 days post- OVA-specific T cells transfer) showed a dramatic reduction (∼50%) however, five days later a clear recovery of this population occurred in mice fed SC (Figs 2B,S3C). Mice fed HFD displayed reduced numbers of Ly6C^lo^ macrophages but in sharp contrast these animals did not display the capacity to re-populate the liver with Ly6C^lo^ macrophages (Fig. S3C). In terms of Ly6C^hi^ macrophages, one of the expected sources of restorative Ly6C^lo^ macrophages, HFD animals showed lower but not significant numbers as compared to SC-fed mice on day 10 post- T cell transfer as well (23 ± 3 vs 31 ± 2.5, p = 0.07) (Fig. S3C).

Prior to OVA-T cells transfer, we identified few neutrophils present in the liver of SC fed mice whereas animals fed HFD exhibited higher numbers of neutrophils than SC mice (0.7 ± 0.1vs 0.1 ± 0.07, respectively) (Figs 2C, S3B). As cholangitis progressed, increasing numbers of neutrophils were observed in both groups however greater infiltration of neutrophils was consistently seen in obese OVAbil mice reaching the maximum on day 10 post-transfer (HFD 18 ± 5.6 vs SC 5.1 ± 2) (Figs 2C, S3B). In addition to the heightened infiltration of neutrophils in the liver of obese mice, these cells exhibited a different phenotype lacking surface expression of CCR1 and CCR5 compared to neutrophils recovered from SC-fed OVAbil mice. Neutrophils from SC mice (∼50%) were CCR1^+^ CCR5^+^ double positive whereas most of the neutrophils from HFD animals (70%) lacked both chemokine receptors (Fig. S5).

### Obesity contributes to increased infiltration of CD8^+^ T cells

To initiate and amplify inflammatory diseases, infiltration of cells with cytotoxic potential such as CD8^+^ T cells is often required. Thus, we aimed to determine the extent of infiltration during experimental PSC in obese mice. Prior to biliary damage, there were low numbers of CD4^+^ and CD8^+^ T cells within the liver with a clear CD4^+^ T cell predominance (Fig. S6C). Five days after OVA-specific T cell transfer, a clear expansion of both CD4^+^ and CD8^+^ T cells was found (Fig. S6C). By day 10, the number of CD8^+^ T cells had tripled compared to CD4^+^ T cells in the HFD group whereas the number of CD8^+^ T cells was only slightly higher than the CD4^+^ T cells from the SC group (Fig. 2D).

Prior to the induction of biliary damage, we observed a significant depletion of CD4^+^ NKT but not CD4^−^ CD8^−^ (double negative) NKT cells resulting from the obesogenic diet. Phenotyping of liver cells in mice fed SC showed ∼21% of lymphocytes were CD4^+^ NKT cells, in contrast, same analysis showed that obese mice had ∼10% of CD4^+^ NKT cell population. (Fig. S6B). Moreover, induction of portal inflammation by transferring OVA-specific T cells led to the progressive loss of CD4^+^ NKT cells over the time with an exaggerated phenotype in obese mice leading to a virtual depletion at the peak of disease compared to SC fed mice (Fig. 2A).

### Obese mice displayed exacerbated production of inflammatory chemokines and cytokines

**Figure 3:**
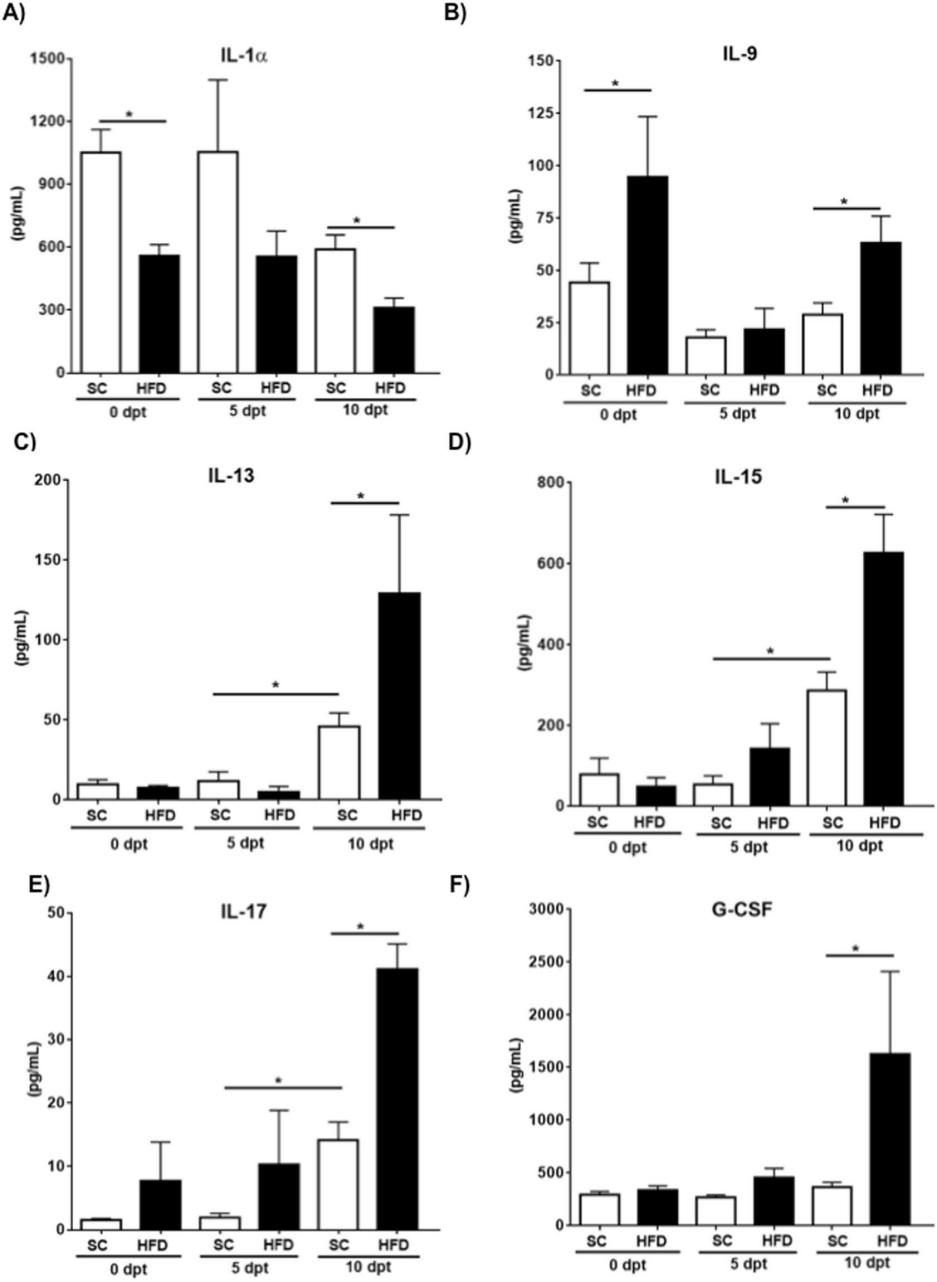
HFD fed mice with biliary damage displayed higher circulating levels of inflammatory mediators. Blood samples were collected before and at indicated times after induction of biliary damage to assess levels of cytokines by means of a pre-determined 31-analyte mouse array. Levels of IL-1α (**A**), IL-9 (**B**), IL-13 (**C**), IL-15 (**D**), IL-17 (**E**) and G-CSF (**F**) are shown. Data are mean ± SE from 2–3 independent experiments (n = 7–10) *p < 0.05 when compared to diet and time matched counterparts. dpt; days post transfer.

**Figure 4:**
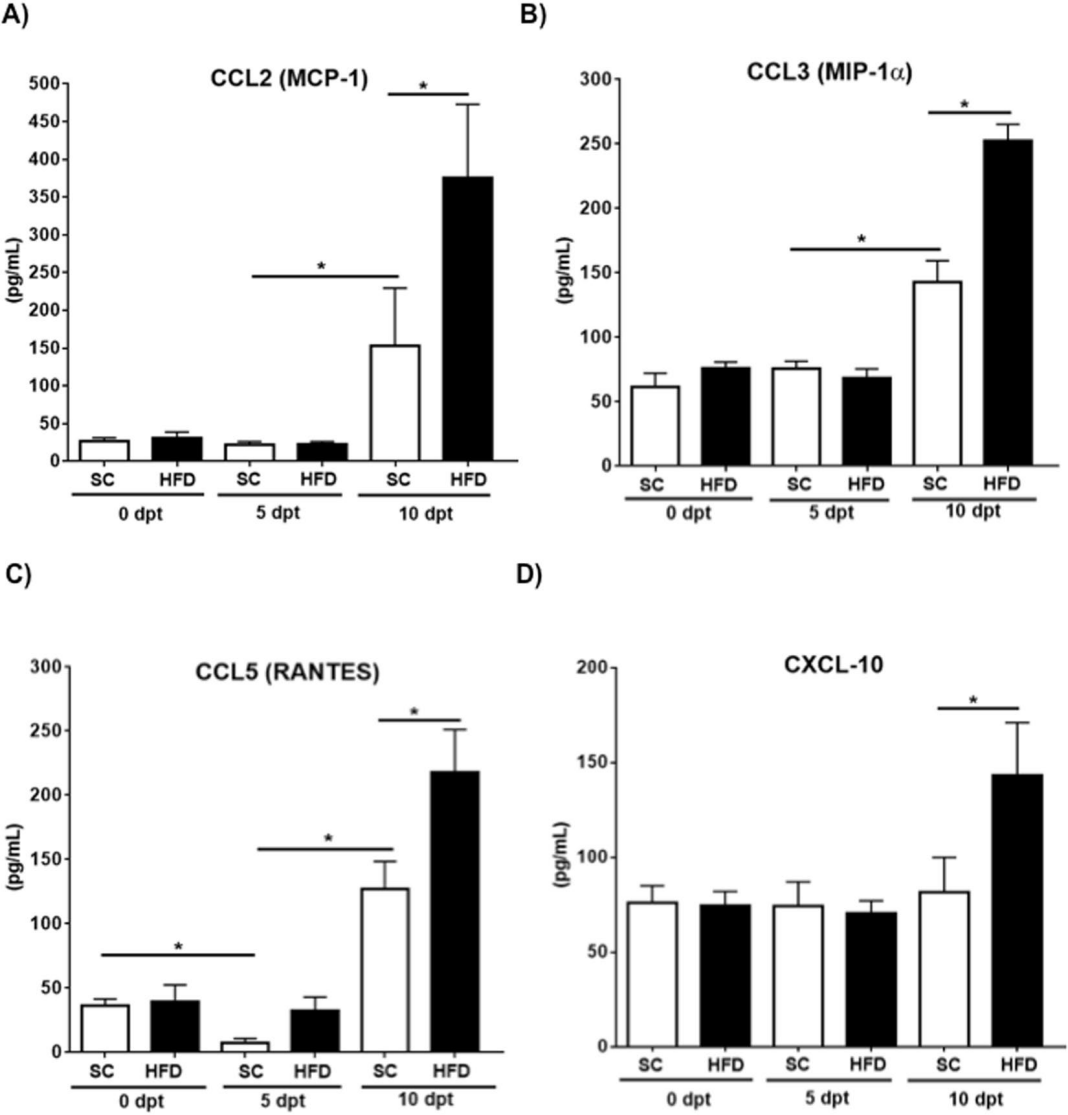
High levels of chemokines are detected at the peak of the disease, which is exaggerated in obese mice. Serum samples were collected at indicated time points and chemokine production was determined. Bar graphs show levels of CCL2, CCL3, CCL5 and CXCL-10 in sera from different experimental groups. *p < 0.05 when compared to time matched counterparts. dpt; days post transfer.

Inflammatory cytokines and chemokines have been shown to contribute to liver pathology whereas pharmacological blockade of IL-17 or transgenic mice deficient in IL-12 result in the reduction of liver damage and fibrosis^18,19^. To gain insight into changes in cytokines and chemokines resulting from diet-induced obesity in mice with experimental PSC, blood samples were collected and assayed to measure multiple analytes simultaneously. We detected increased production of IL-9, IL-13, IL-15, IL-17 and the granulopoietic factor G-CSF in mice fed the HFD on day 10 compared with mice fed SC (Fig. 3B–F). Enhanced levels of TNFα were detected in mice with PSC however no significant difference between diet groups was found. We observed that obese mice had supressed production of IL-1α prior to biliary damage induction and trended towards statistical significance on day 5 compared to SC fed mice (Fig. 3A). On day 10, both groups showed decreased secretion of IL-1α with a more pronounced effect in obese animals (Fig. 3A). While biliary damage triggered the release of CCL2, CCL3 and CCL5 on day 10 post-transfer, mice fed HFD showed exaggerated secretion of these chemokines as well as CXCL-10 (Fig. 4A–D).

### Interleukin-15 plays a critical role in exacerbated PSC in obese mice

**Figure 5:**
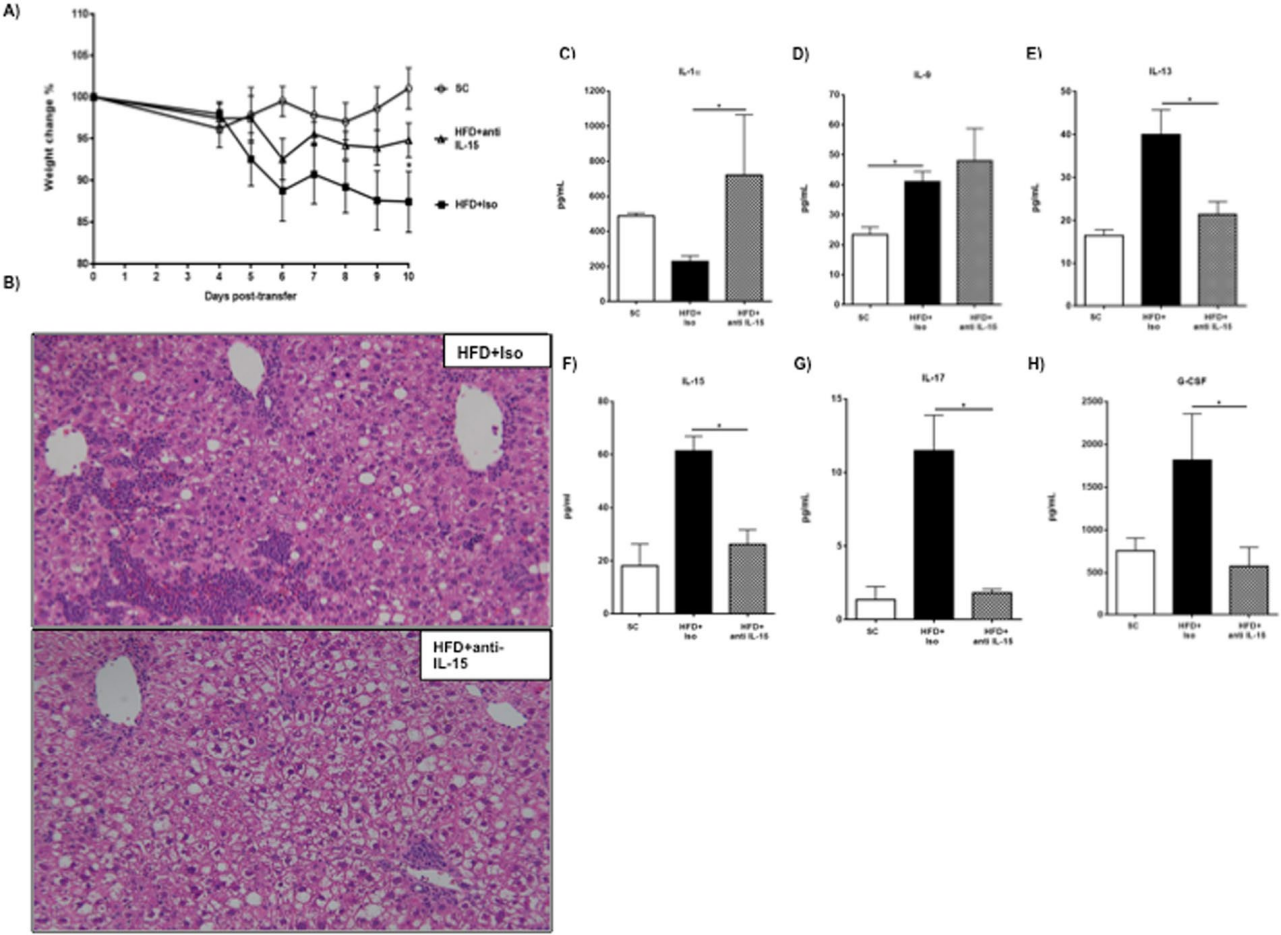
Immuno-neutralization of IL-15 reverted biliary injury in obese mice. After twelve weeks feeding mice with either SC or HFD mice were transferred with OVA-specific T cells. Mice fed HFD were randomly caged and one group received isotype antibodies (Iso) whereas second groups received anti-IL-15 blocking antibodies (anti-IL-15). Monitoring of weight changes started on day 4 before the first injection of either Iso or anti-IL-15 (**A**). On autopsy day, tissue samples were collected and histopathology was assessed in H&E sections. Representative image of obese, isotype-treated mice show an extensive inflammatory infiltrate in (**B**). Blood samples were obtained by cardiac puncture and assayed for cytokine output. Levels of IL-1α (**C**) and damage-associated IL-9 (**D**), IL-13 (**E**), IL-15 (**F**), IL-17 (**G**), G-CSF (**H**) are shown. Data are representative of 2 experiments (HFD+ Iso, n = 7) (HFD+ anti-IL-15, n = 9) magnification 20×. dpt; days post transfer.

A central role for CD8^+^ T cells driving experimental PSC in the OVAbil mouse model has been described^12^. In line with this observation, we identified increased numbers of CD8^+^ T cells at the peak of cholangitis that correlated with elevated circulating levels of IL-15 in obese OVAbil mice. To determine the consequence of increased IL-15, we administered an IL-15 blocking antibody to HFD mice starting on day 4 post-T cell transfers based on our previous observation that CD8+ T cells begin to expand around day 5 post adoptive transfer (Fig. 2D). Similar to our previous observation, obese OVAbil mice lost significantly more weight than SC mice after adoptive T cell transfer however IL-15 neutralization tended to prevent the majority of weight loss in obese animals only reaching statistical difference on day 10 (Fig. 5A). In regards of histological damage, HFD mice receiving OVA-specific T cells and isotype control antibody showed periportal and parenchymal inflammatory infiltrate and this was significantly improved to only periportal infiltration with visibly reduced size when we used neutralizing IL-15 antibody treatment (Fig. 5B). Moreover, blockade of IL-15 modified the plasma cytokine profile compared to obese animals treated with the isotype control. Cytokine profiling revealed important findings, for example IL-1α levels, which were low upon liver damage, notably increased when mice were treated with anti-IL-15 antibody (Fig. 5C). Also, except for IL-9, cytokines including IL-13, IL-17 and G-CSF were supressed by anti-IL-15 treatment (Fig. 5E–H). Inflammatory chemokines were also impacted by neutralization of IL-15, this latter resulted in inhibition of CCL2, CCL3 and CXCL10 secretion.

**Figure 6:**
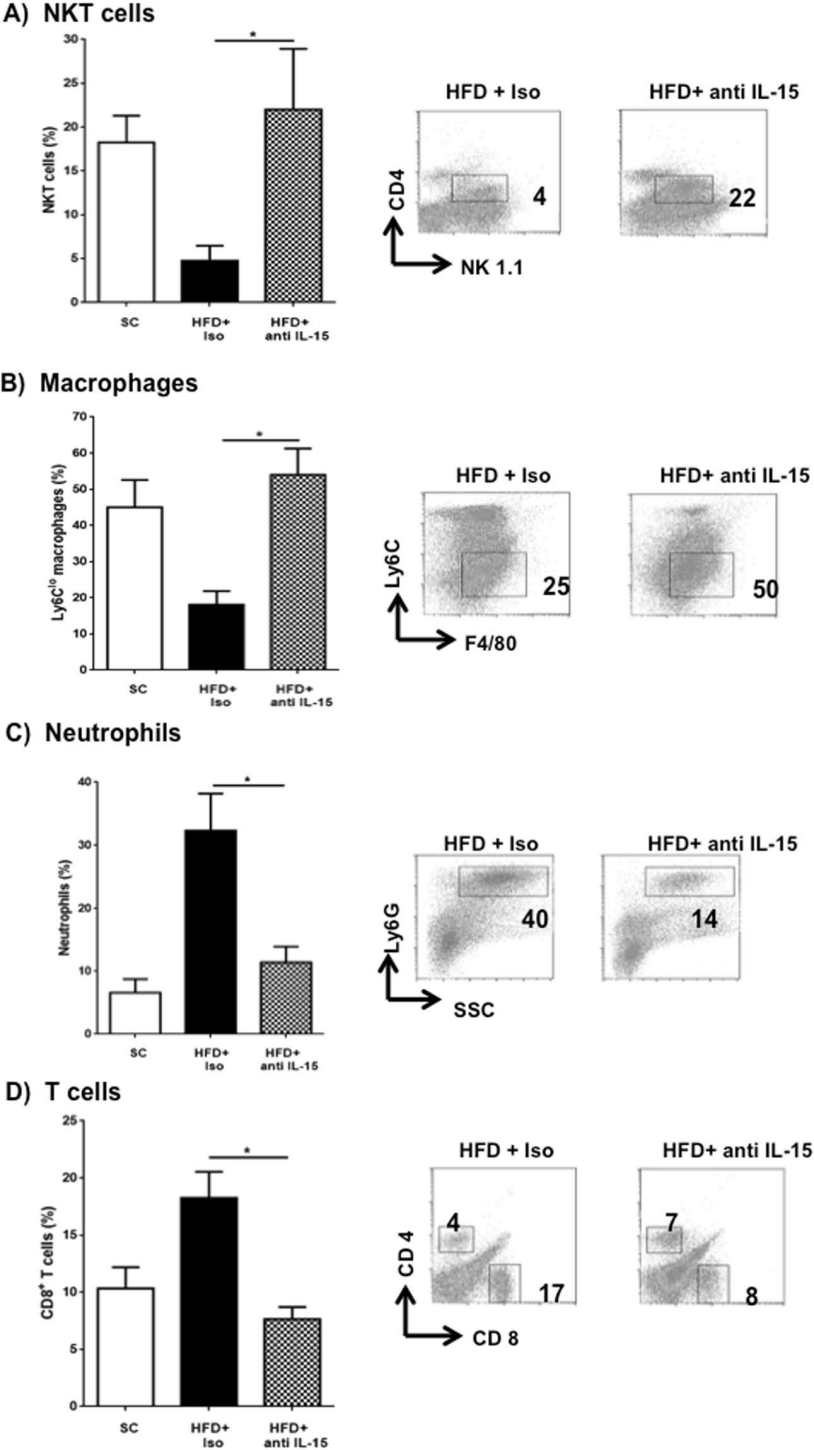
Anti-inflammatory cell populations re-appear after blocking IL-15 whereas injury-promoting cells decrease. On day 10, liver tissues from experimental groups were homogenized and white cells obtained by percoll gradient to perform flow cytometry following gating strategy described in methods. Panels (A) and (B) Graphs depict % of CD4^+^NKT cells and restorative macrophages (CD11b^+^ F4/80^+^ Ly6^lo^), respectively alongside representative plots. Neutrophils (Ly6G^hi^ SSC^hi^) and cytotoxic T cells (CD3^+^CD8^+^) % and representative plots are shown in panels (C) and (D) respectively. Data are from 2 experiments (SC, n = 4) (HFD+ Iso, n = 7) (HFD+ anti-IL-15, n = 9) where *p < 0.05. dpt; days post transfer.

Flow cytometry analysis revealed that blocking IL-15 in obese mice led to the recovery of previously diminished tolerogenic leukocyte populations such as NKT lymphocytes and macrophages (Fig. 6A,B). Ly6C^lo^ macrophages (likely restorative) were prominent whereas the Ly6C^hi^ macrophage population was almost negligible suggesting that IL-15 could be dampening Ly6C^hi^ to Ly6C^lo^ macrophage transition. Also, blockade of IL-15 concomitantly restrained expansion of pathogenic cells like neutrophils and cytotoxic lymphocytes (Fig. 6C and D).

## Discussion

One of the most concerning outcomes of the obesity pandemic is the increased incidence of co-morbidities including diabetes, cardiovascular disease and liver disease^20^. Recent research has revealed that obesity is associated with more severe development of gastrointestinal disease^20^. The impact of obesity on certain liver pathologies including the development of PSC has not been explored to date.

Our clinical observation identified a group of PSC patients with a strong association between higher BMI and more advanced fibrosis stage suggesting that immune changes accompanying adipocyte hyperplasia and hypertrophy might be accelerating the transition from inflammatory to fibrotic stages of liver disease^9^. To investigate the link between obesity and more severe PSC we examined the development of experimental PSC in obese mice. We confirmed that mice fed HFD had aggravated portal tract damage along with exacerbated biliary damage as gauged by periportal inflammation, portal space reduction and absence (assessed in the majority of portal tracts) of BDs compared to standard chow fed mice.

Expansion of CD8^+^ T cells has been associated with increased disease severity in this model of immune-mediated cholangitis^12^. As expected we observed expansion of CD8^+^ T cells upon transfer of pathogenic T cells in non-obese mice. However, mice fed the HFD had an uncontrolled expansion of the same population that correlated with higher levels of IL-15 in the serum. Interleukin-15 is produced by cells from both hematopoietic and non-hematopoietic origin and has been linked to obesity and liver disease^21^. On one hand, IL-15 can exert anti-adipogenic functions and may promote energy expenditure^22–24^. However, IL-15 may also lead to the accumulation of lipid within hepatocytes and contribute to the development of non-alcohol fatty liver disease (NAFLD)^25^. Therapeutic blockade of IL-15 in obese mice with cholangitis resulted in attenuated histological damage, lower numbers of infiltrating neutrophils and CD8^+^ T cells and reduced levels of proinflammatory cytokines and chemokines including IL-13, IL-17, C-GSF, CCL2, CCL3 and CXCL10. In PSC, IL-15 may represent the inflammatory switch driving exaggerated biliary damage. While IL-15 levels never disappeared after blockade this is most likely due to constant release in response to rapid clearance and short-term lifespan of the cytokine^21^. Continued examination of the role of IL-15 along with other pathological cytokines as potential therapeutic targets is required.

Cytokines are important drivers in liver inflammation and may contribute to tissue injury^18^. The cytokine profile of our experimental groups was a mixed phenotype representing both a Th2 and Th17 response consisting of IL-13 and IL-17 with biliary damage induced by pathogenic cholangiocyte-specific T cells. Although it is conventionally thought that IL-13 and IL-17 are mutually regulated, this cytokine profile was exaggerated in obese animals showing that both Th2 and Th17 responses may co-exist^26,27^. In our model it is possible that numerous cytokines are acting synergistically to cause a more severe phenotype of experimental PSC. Interestingly, attenuation of GI tract diseases has been reported in the absence of either cytokine^28,29^. It is tempting to speculate that an IL-13^+^ IL-17^+^ double positive T helper subpopulation with enhanced pathogenicity emerges during biliary injury in obese animals. Studies addressing this possibility are currently taking place in our laboratory. We also found higher levels of IL-9 in obese mice suggesting that IL-9 may be acting as an additional factor given this Th2-like cytokine has been found to promote inflammation in the GI tract^30–32^.

Interestingly, levels of IL-1α inversely correlated with obesity and biliary injury and as disease severity increased, IL-1α levels tended to decrease. A clear explanation for this phenomenon remains controversial as IL-1α is thought to display similar inflammatory features to IL-1β^33^ and conflicting reports show a positive relationship between obesity and IL-1α^34,35^ while others show an inverse relationship^36^ or no clear correlation^37,38^. When we neutralized IL-15, levels of IL-1α were restored and this was associated with attenuated cholangitis.

Macrophages have been described as a key cell type potentially contributing to both the promotion and resolution of liver inflammation and tissue injury. One paradigm stratifies macrophages based on Ly6C expression where Ly6C^hi^ macrophages may promote inflammation in early stages whereas Ly6C^lo^ occur later and may limit inflammation^17,39^. Although we observed diminished numbers of the latter population in early phase of disease, non-obese mice were able to recover Ly6C^lo^ macrophages in the later phase of disease while obese mice did not recover this Ly6C^lo^ macrophage population even when Ly6C^hi^ F4/80^+^ cells were not significantly affected. This suggests that macrophage proliferation rather than infiltration may be responsible for the inability to recover putative restorative Ly6C^lo^ macrophages. Critically, blocking of IL-15 resulted in the reappearance of Ly6C^lo^ macrophages in the liver tissue of obese mice and the number of Ly6C^lo^ macrophages in obese mice surpassed the number of Ly6C^lo^ macrophages in non-obese animals suggesting that in absence of IL-15 infiltrating macrophages are able to rapidly transition into restorative Ly6C^lo^ macrophages.

Although we did not observe any fibrosis in this model of PSC, obese mice exhibited enhanced expression of TIMP-1, a pro-fibrotic gene emerging as a consequence of obesity^40,41^ and promoted by IL-13 and IL-17^42,43^ as well as high levels of pro-fibrotic chemokines such as CCL3 and CCL5^44–46^. A role for CCR5^+^ neutrophils sequestering CCL3 and CCL5 during the resolution phase of inflammation has been shown to support decreased fibrosis and a return to homeostasis^47^. Interestingly, in this current study the mice with obesity and cholangitis were found to have predominately CCL5^−^ neutrophils in the liver as well as higher serum levels of CCL3 and CCL5. This data suggests that the onset of fibrosis in PSC during obesity may in part be influenced by the balance between pro-inflammatory and pro-resolution neutrophils. Therefore, future studies longer in duration will evaluate the potential of accelerated fibrosis in obese animals following a strong inflammatory reaction.

In summary, diet-induced obesity results in the reduced presence of tolerogenic-like cell populations in the liver. This in turn prevents the suppression of subsequent inflammatory challenges and primes cells to over-produce IL-15. Higher levels of IL-15 subsequently exaggerates severity in our murine model of antigen-driven liver disease. As our knowledge of PSC continues to develop there is potential to generate novel mouse models that may enhance our understanding of this chronic, immune-mediated disease. Future studies evaluating the potential to neutralize the detrimental effects of cytokines such as IL-15 may represent a novel treatment strategy for patients with PSC.

## Electronic supplementary material

Supplementary Figures
