# Supplementary material for: Neutralization of IL-15 abrogates experimental immune-mediated cholangitis in diet-induced obese mice
Source: Sci Rep. 2018 Feb 15;8:3127. doi: 10.1038/s41598-018-21112-7 (PMC5814438; doi:10.1038/s41598-018-21112-7)
Supplement: Supplementary file 1 — Supplementary Figures [file 41598_2018_21112_MOESM1_ESM.pdf]

Supplementary Figures

Neutralization of IL-15 abrogates experimental immune-mediated cholangitis in diet-induced obese mice

José L. Reyes[1, 2], Danielle T. Vannan[1,6], Tina Vo[1], Aliya Gulamhusein[3], Paul L. Beck[1], Raylene A. Reimer[4,5] and Bertus Eksteen[6,*]

[1]Snyder Institute for Chronic Diseases, Cumming School of Medicine, University of Calgary, Canada

[2]Laboratorio de Inmunología Experimental y Regulación de la Inflamación Hepato-Intestinal, UBIMED, FES Iztacala UNAM, Estado de México, México.

[3]Genomic Hepatobiology Laboratory, Mayo Clinic, Rochester, Minnesota, USA.

[4]Department of Biochemistry & Molecular Biology, Cumming School of Medicine, University of Calgary, Canada.

[5]Faculty of Kinesiology, University of Calgary, Canada.

[6]Aspen Woods Clinic, Calgary, Alberta, Canada.

***Senior and corresponding author:**

Dr. Bertus Eksteen
Director, Calgary PSC Clinic
Aspen Woods Clinic
8561 – 8A Ave SW, Suite 2314
Calgary, Alberta, Canada
T3H 0V5
Tel. 403-455-7872
bertus@aspenwoodsclinic.com

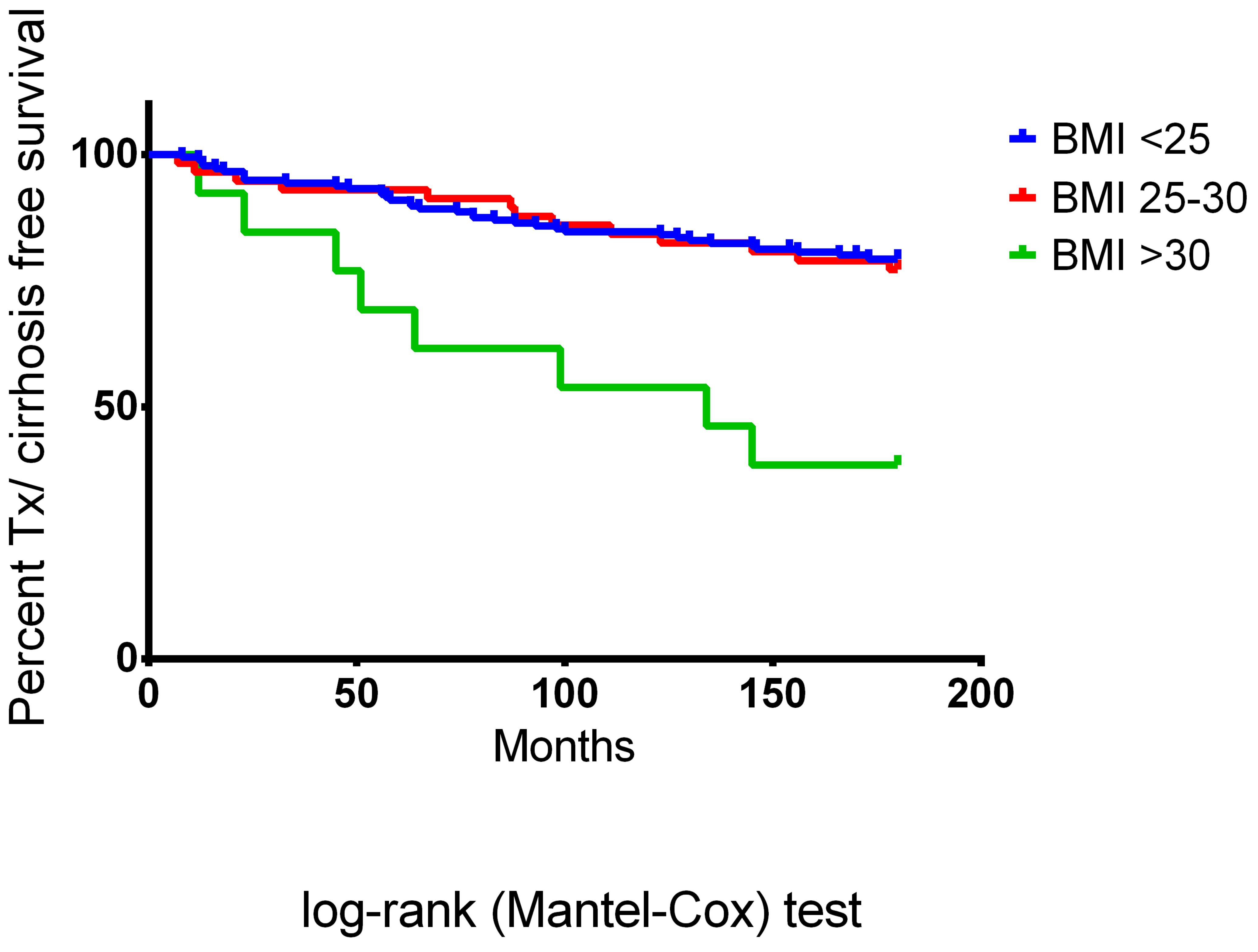
Percent Tx/ cirrhosis free survival
100
50
0
0
50
100
150
200
Months
BMI <25
BMI 25-30
BMI >30
log-rank (Mantel-Cox) test

A)

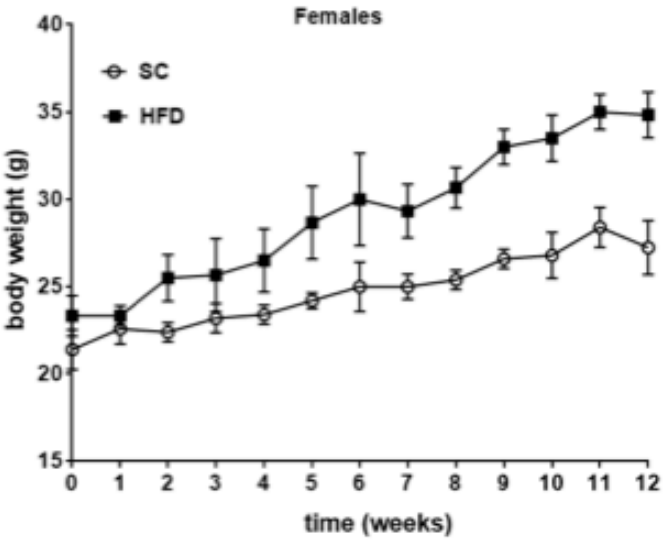
Females
SC
HFD
body weight (g)
time (weeks)
Males
HFD
SC
body weight (g)
time (weeks)

B)

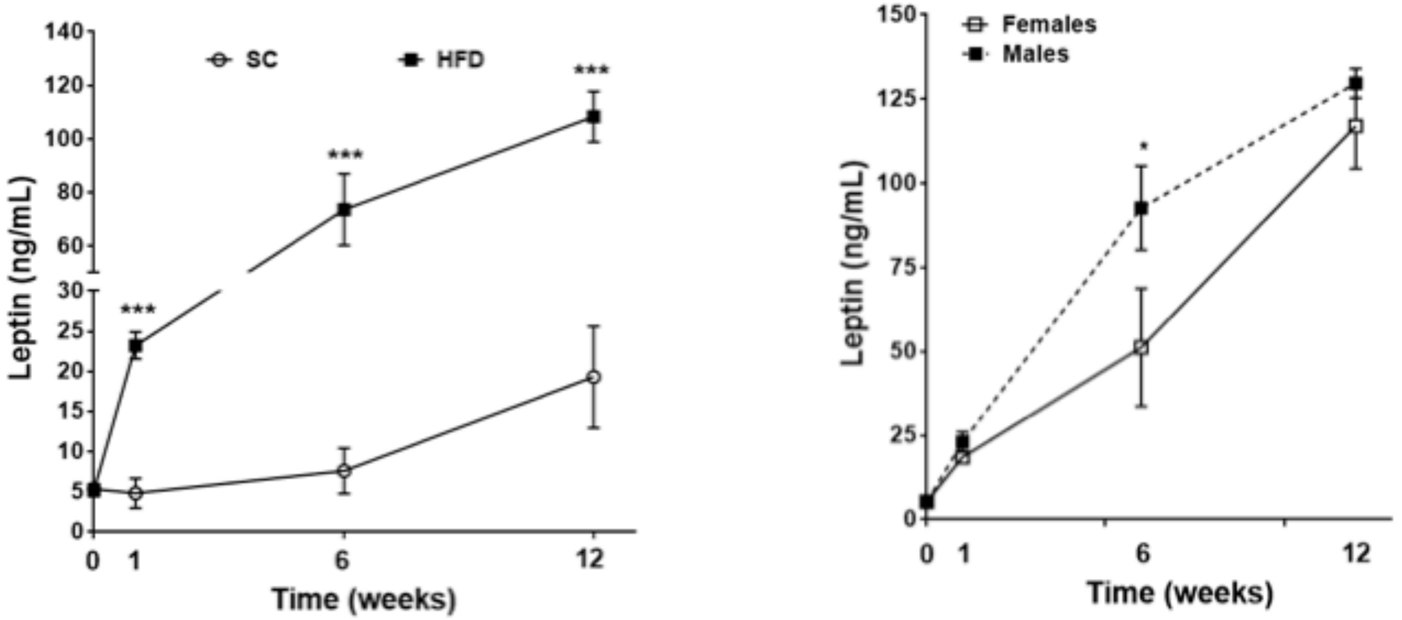
SC
HFD
***
***
***
Leptin (ng/mL)
Time (weeks)
Females
Males
*
Leptin (ng/mL)
Time (weeks)

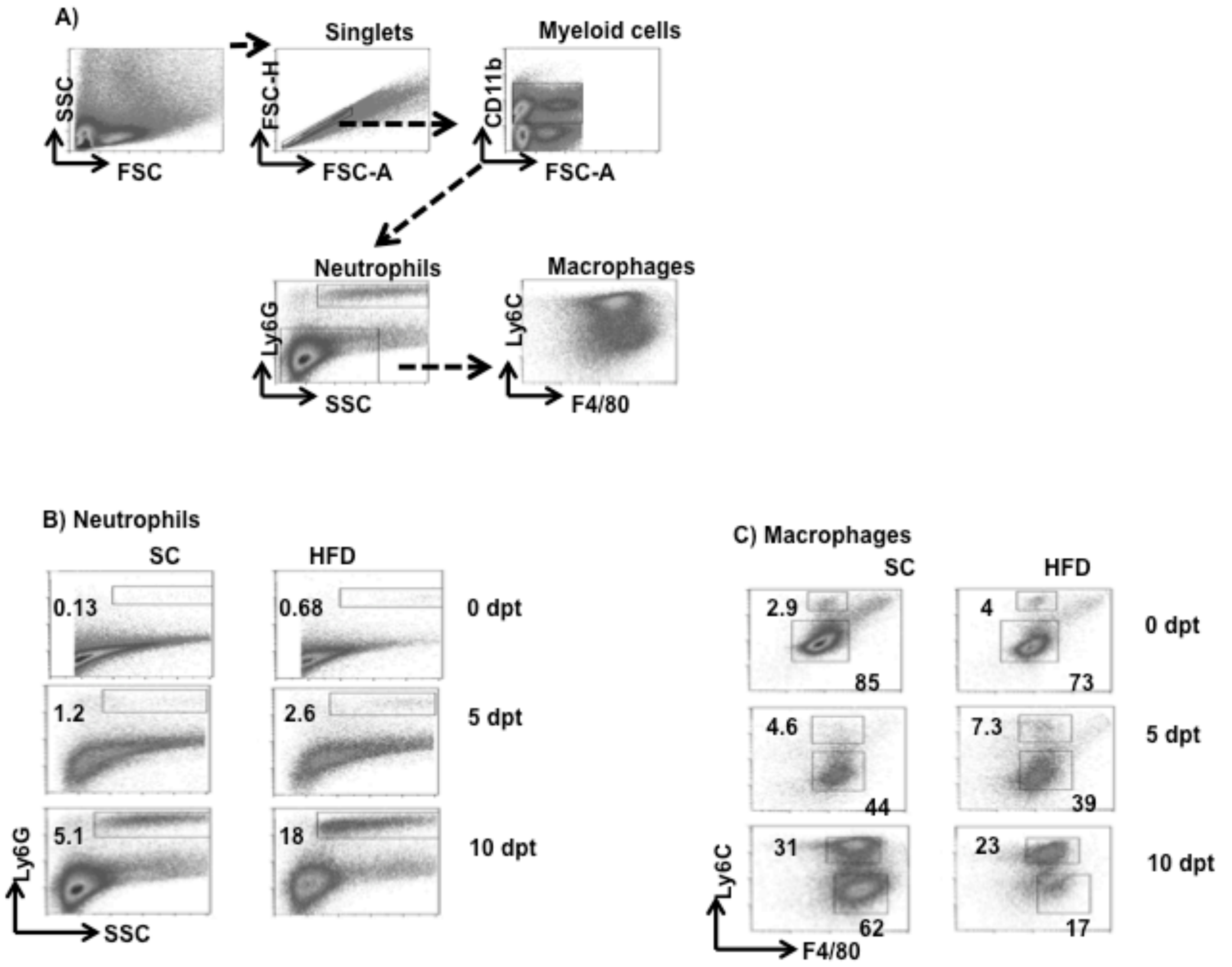
A)
Singlets
Myeloid cells
SSC
FSC
FSC-H
FSC-A
CD11b
FSC-A
Neutrophils
Macrophages
Ly6G
SSC
Ly6C
F4/80
B) Neutrophils
SC
HFD
0.13
0.68
0 dpt
1.2
2.6
5 dpt
5.1
18
10 dpt
Ly6G
SSC
C) Macrophages
SC
HFD
2.9
85
4
73
0 dpt
4.6
44
7.3
39
5 dpt
31
62
23
17
10 dpt
Ly6C
F4/80

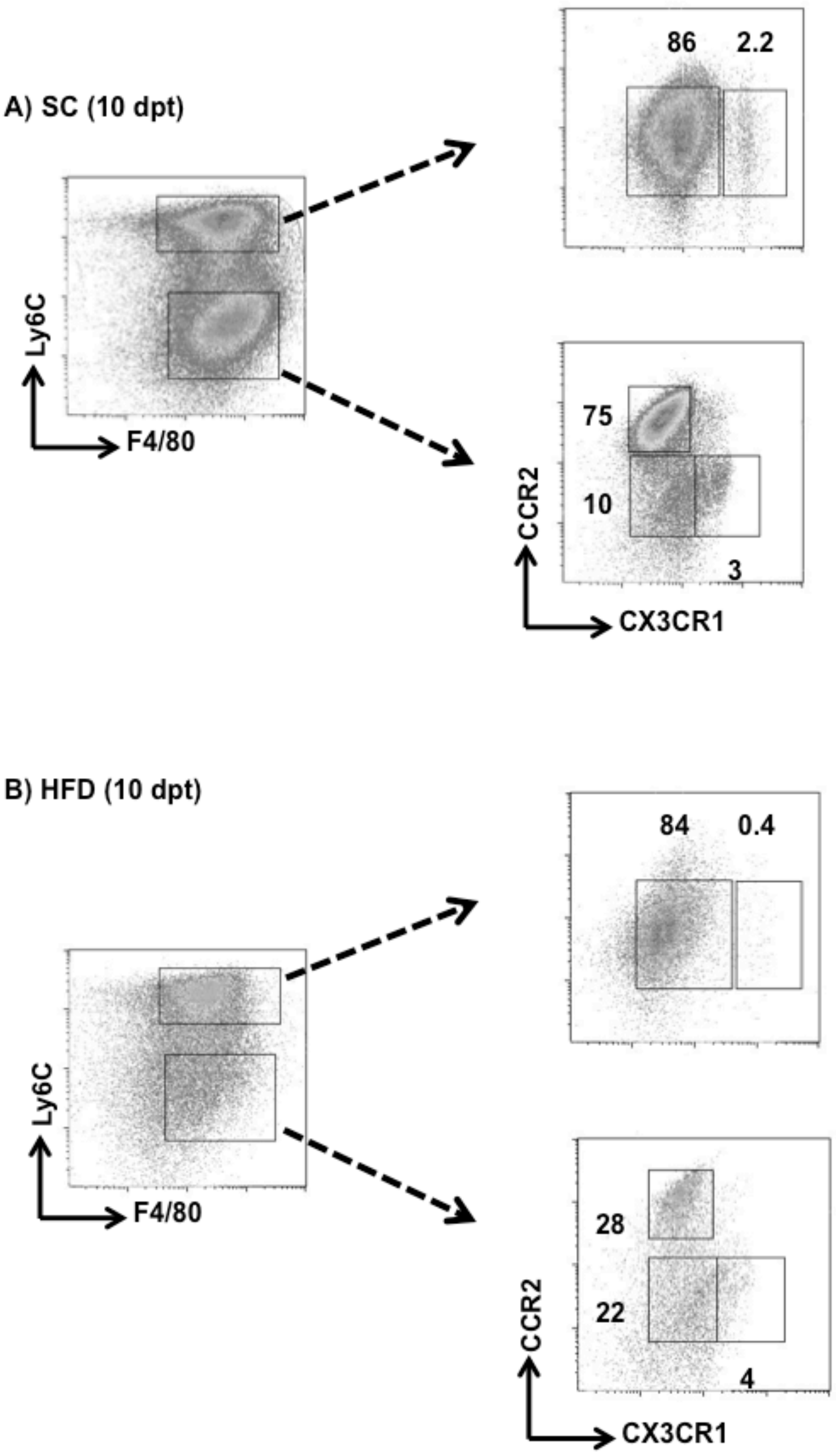
A) SC (10 dpt)
Ly6C
F4/80
86
2.2
75
10
3
CCR2
CX3CR1
B) HFD (10 dpt)
Ly6C
F4/80
84
0.4
28
22
4
CCR2
CX3CR1

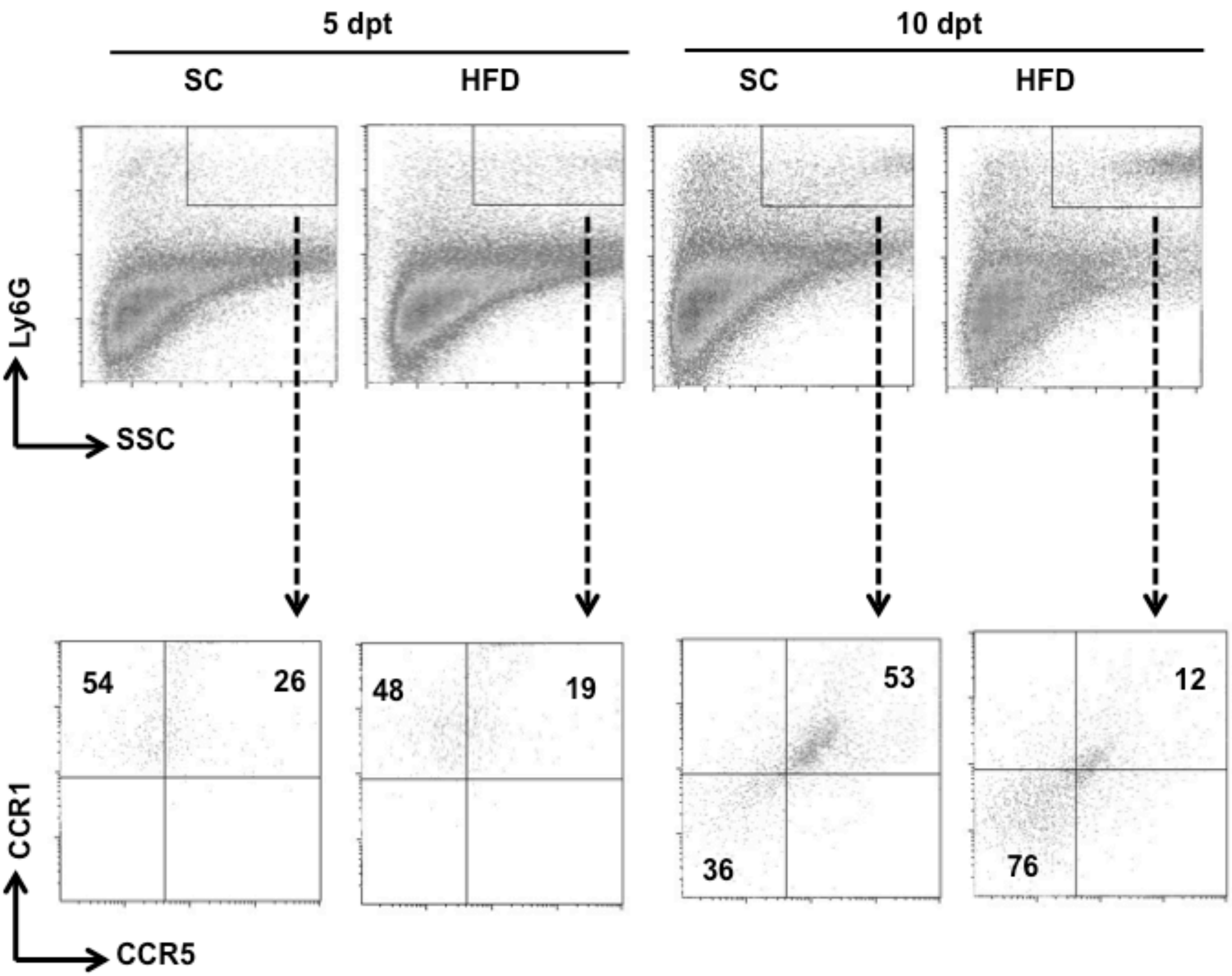
5 dpt
10 dpt
SC
HFD
SC
HFD
Ly6G
SSC
54
26
48
19
53
36
12
76
CCR1
CCR5

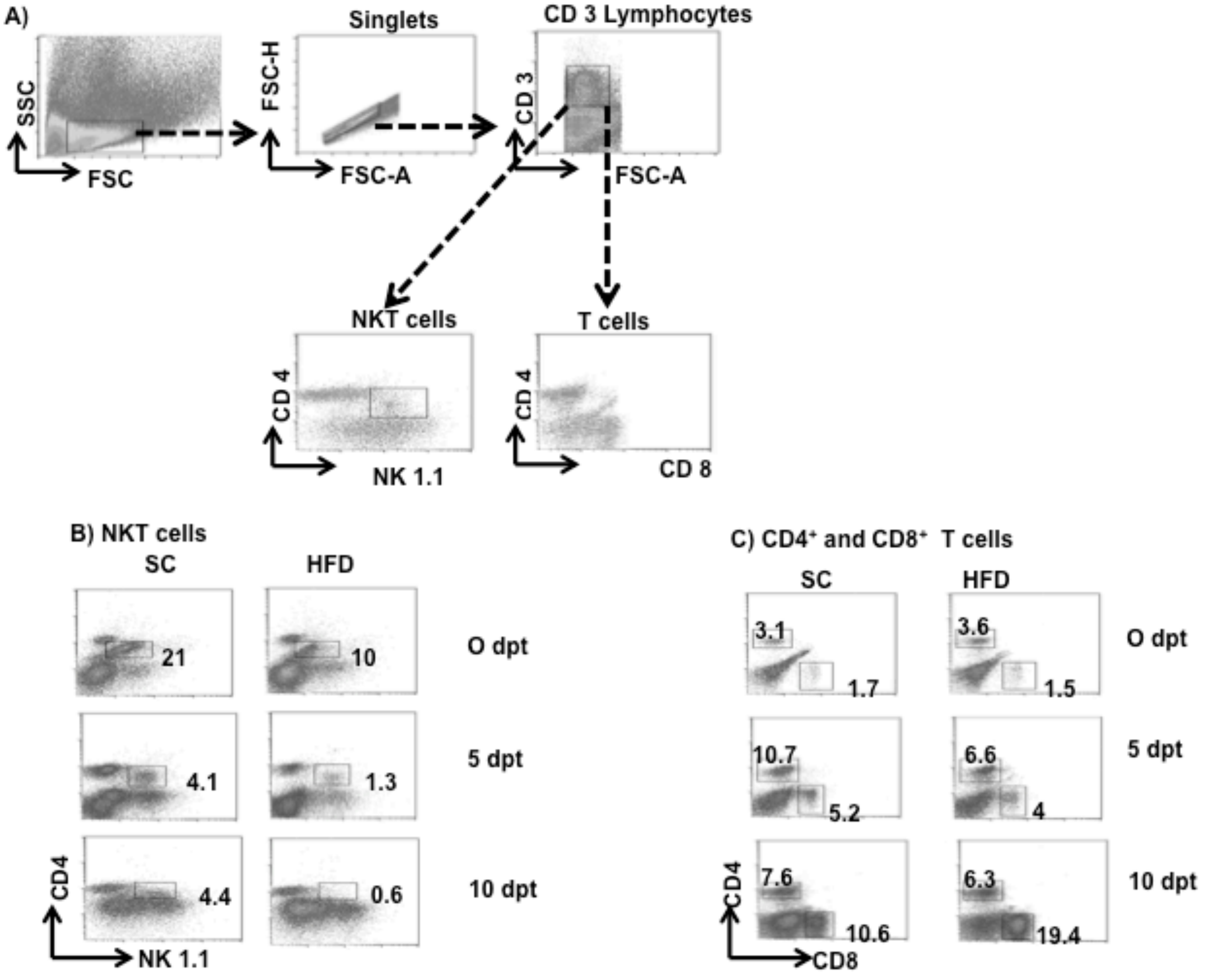
A)
Singlets
CD 3 Lymphocytes
SSC
FSC
FSC-H
FSC-A
CD 3
FSC-A
NKT cells
T cells
CD 4
NK 1.1
CD 4
CD 8
B) NKT cells
SC
HFD
21
10
O dpt
4.1
1.3
5 dpt
4.4
0.6
10 dpt
CD4
NK 1.1
C) CD4+ and CD8+ T cells
SC
HFD
3.1
1.7
3.6
1.5
O dpt
10.7
5.2
6.6
4
5 dpt
7.6
10.6
6.3
19.4
10 dpt
CD4
CD8
